# Molecular Characterization of KPC-2-Producing *Enterobacter cloacae* Complex Isolates from Cali, Colombia

**DOI:** 10.3390/antibiotics10060694

**Published:** 2021-06-10

**Authors:** Aura Falco, Daniela Guerrero, Isabella García, Adriana Correa, Sandra Rivera, María Beatriz Olaya, Carlos Aranaga

**Affiliations:** 1 Grupo de Investigación en Microbiología, Industria y Ambiente (GIMIA), Facultad de Ciencias Básicas, Universidad Santiago de Cali, Cali 760035, Colombia; daniela.guerrero00@usc.edu.co (D.G.); isabella.garcia01@usc.edu.co (I.G.); adriana.correa00@usc.edu.co (A.C.); sandra.rivera04@usc.edu.co (S.R.); 2 Clínica Imbanaco, Cali 760042, Colombia; 3 Laboratorio de Salud Pública Departamental, Secretaria Departamental de Salud del Valle del Cauca, Gobernación del Valle del Cauca, Cali 760045, Colombia; bolayag@gmail.com; 4 Grupo de Investigación en Química y Biotecnología (QUIBIO), Facultad de Ciencias Básicas, Universidad Santiago de Cali, Cali 760035, Colombia; carlos.aranaga00@usc.edu.co

**Keywords:** carbapenems, resistance, KPC, *Enterobacter cloacae* complex, Colombia, ST510

## Abstract

The *Enterobacter cloacae* complex is an emerging opportunistic pathogen whose increased resistance to carbapenems is considered a public health problem. This is due to the loss of efficacy of beta-lactam antibiotics, which are used as the first treatment option in the management of infections caused by Gram-negative bacteria. The objective of this study was to perform the molecular characterization of 28 isolates of the *E. cloacae* complex resistant to cephalosporins and carbapenems isolated between 2011 and 2018 from five hospitals located in the municipality of Santiago de Cali, Colombia. Molecular detection of *bla*_KPC_, *bla*_VIM_, *bla*_NDM_ and *bla*_OXA-48-like_ genes was performed on these isolates and the genetic relationship between the isolates was assessed using multilocus sequence typing (MLST). Forty-three percent of the isolates carried the *bla*_KPC-2_ gene variant. MLST showed high genetic diversity among isolates, the most frequent being the sequence type ST510 with a frequency of 50%. The identification of the genes involved in carbapenem resistance and dispersing genotypes is an important step toward the development of effective prevention and epidemiological surveillance strategies in Colombian hospitals.

## 1. Introduction

The Enterobacter cloacae complex includes several species (*Enterobacter asburiae*, *Enterobacter carcinogenus*, *Enterobacter cloacae*, *Enterobacter hormaechei*, *Enterobacter kobei*, *Enterobacter nimipressuralis*, *Enterobacter ludwigii* and *Enterobacter mori*) [1,2] that are opportunistic pathogens, which belong to the Family Enterobacteriaceae and are considered one of the causal agents of healthcare-associated infections (HAIs) both internationally [3,4,5,6] and nationally [7,8]. Because carbapenems are used to treat infections caused by extended-spectrum beta-lactamase (ESBL)-producing *E. cloacae* complex, the increase in carbapenem resistance in this species is a matter of concern. Studies worldwide report that carbapenem resistance in this species is mainly associated with the production of *Klebsiella pneumoniae* carbapenemases (KPCs) [9,10,11,12,13,14]; however, there are other resistance mechanisms associated with this phenotype such as changes in permeability of cell membrane, efflux pumps and hyper-expression of AmpC [15,16]. The National Institute of Health in Colombia reports that, between 2012 and 2018, 90% of *Enterobacteriaceae* isolates causing HAIs were resistant to carbapenems [17]. Of these, 17% are *E. cloacae* complex isolates, being the second most frequently reported. Ninety four percent of these were carbapenems resistant and 67% were KPC-producers, followed by VIM with 3.5% and NDM with 2.2%. This highlights the importance of KPC-producing *E. cloacae* complex in Colombia [8,17]. Although other studies in Colombia have reported carbapenemase-producing *E. cloacae* complex isolates in the country [7,18,19], there is no updated data specific to the region. Hence, the molecular characterization of KPC-producing *E. cloacae* complex isolated from five tertiary care hospitals located in the municipality of Santiago de Cali from 2011 to 2018 has been performed in the present study.

## 2. Results

### 2.1. Collection of E. cloacae Complex Clinical Isolates

**Figure 1:**
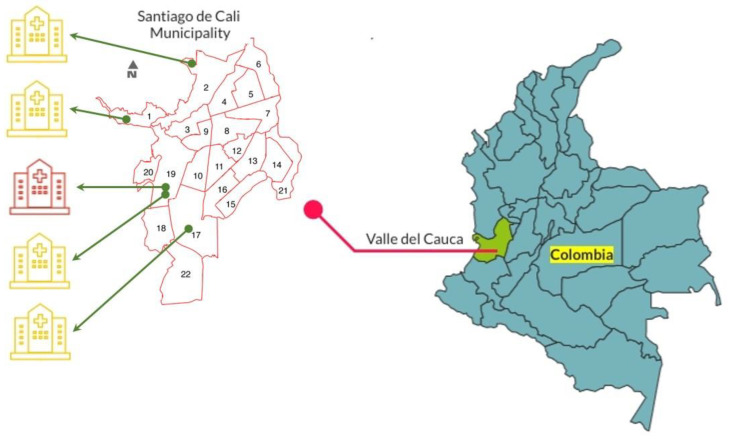
Location of the hospitals from which *E. cloacae* complex isolates were obtained. Four of them are private (in yellow) and one is public (in red); all are in four communes of the municipality of Santiago de Cali, Department of Valle del Cauca, Colombia (Made through https://www.visme.co/, accessed on 29 March 2021).

According to the selection criteria (see Materials and Methods), 28 clinical isolates of *E. cloacae* complex were selected and showing a phenotypic profile of total or intermediate resistance to third and fourth generation cephalosporins and to at least one carbapenem (Table 1). Twenty-one percent (6/28) were isolated from a public tertiary care hospital located in commune 19 (Figure 1, Table 1). The remaining 79% (22/28) were isolated from four private tertiary care hospitals, distributed as follows: 54% (15/28) from clinic 1 (commune 19), 14% (4/28) from clinic 2 (commune 17), 7% (2/28) from clinic 3 (commune 2) and 4% (1/28) from clinic 4 (commune 1) (Figure 1, Table 1). All the communes are in the municipality of Santiago de Cali, Department of Valle del Cauca, in southwest Colombia (Figure 1).

Additionally, Table 1 presents the information related to the clinical isolates (year of isolation and hospital of origin) and the demographic data associated with the samples (age and sex of the patient and site of infection).

### 2.2. Detection of Genes Encoding Beta-Lactamase Enzymes

The *bla*_KPC-2_ gene was detected in 43% (12/28) of the isolates (Table 2). None of them carried *bla*_VIM_, *bla*_NDM_, or *bla*_OXA-48-like_ genes that code for other carbapenemases.

### 2.3. Clinical and Demographic Characteristics of the E. cloacae Complex Isolates Carrying the bla_KPC-2_ Gene

We analyzed the twelve *E. cloacae* complex isolates found to carry the *bla*_KPC-2_ gene, which were isolated from five tertiary care hospitals. Twenty-five percent (3/12) of the isolates were obtained from a public hospital between 2017 and 2018. The remaining 75% (9/12) were obtained from private entities and were distributed as follows: 42% (5/12) from clinic 1 between 2013 and 2017; 17% (2/12) from clinic 2 in 2017 and 8% (1/12) from clinics 3 and 4 in 2017 and 2018, respectively.

The demographic characteristics are summarized in Table 3. Eighty-three percent of infected patients were male and 32% were elderly. The most common site of infection was blood, affecting 42% of patients (Table 3).

### 2.4. Molecular Genotyping of KPC-Producing E. cloacae Complex Isolates

**Figure 2:**
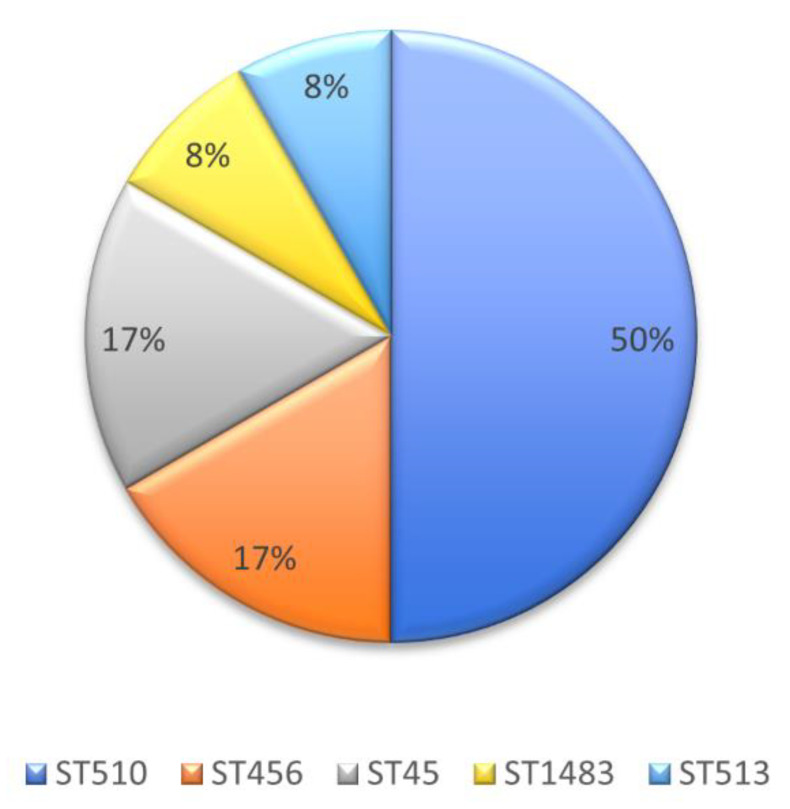
Percentage of sequence types (STs) of the *E. cloacae* complex isolates carrying the *bla*_KPC-2_ gene circulating in the municipality of Santiago de Cali from 2013 to 2018.

The genotyping analysis of the 12 evaluated KPC-producing *E. cloacae* complex isolates yielded five STs, namely ST510 (50%, 6/12), ST45 (17%, 2/12), ST456 (17%, 2/12), ST1483 (8%, 1/12) and ST513 (8%, 1/12) (Figure 2, Table 2).

Two STs were found in two different hospitals. The first one was ST510, which was identified in four isolates from clinic 1 and two isolates from the public entity. Both were in commune 19 (Figure 1), which indicates that ST510 circulated from 2013 to 2018 in the southwest of the municipality of Santiago de Cali. The second ST was ST456, which was found in one isolate from clinic 3 (commune 2) and one isolate from clinic 4 (commune 1) (Figure 1). Therefore, ST456 circulated in the northwest of the city between 2017 and 2018.

The other three STs were found in a single hospital, with one isolate each. Thus, ST1483 was found in clinic 1 (commune 19) in 2014, ST513 in the public entity (commune 19) in 2017 and ST45 in clinic 2 (commune 17) in 2017.

**Figure 3:**
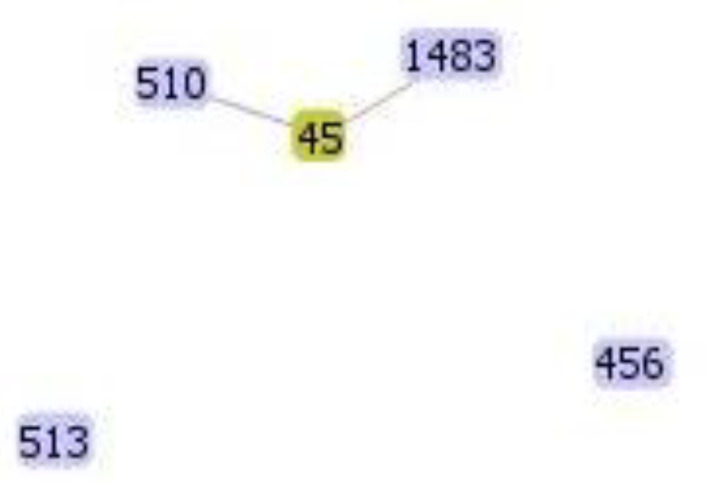
Cluster of the *E. cloacae* complex isolates carrying the *bla*_KPC-2_ gene generated with the goeBURST algorithm.

The results obtained using the goeBURST algorithm indicated high genetic diversity because there were no single, double, or triple locus variants. However, ST510 and ST45 are satellites, because they share three of the seven genes with each other (*dnaA*, *fusA* and *rplB*) (Figure 3, Table 3), whereas ST510 and ST45 share the *rplB* allele with ST1483 (Figure 3, Table 3). Additionally, there were two STs, ST513 and ST456, that remained unclustered, i.e., as singletons (Figure 3, Table 3).

## 3. Discussion

According to our results, 43% (12/28) of the carbapenem-resistant *E. cloacae* complex isolates evaluated in this study carried the variant of the *bla*_KPC-2_ gene. This percentage is lower than that reported by De la Cadena et al. (2017), who also performed a molecular characterization of 28 strains of carbapenem-resistant *E. cloacae* complex isolated between 2009 and 2013 from eight cities in Colombia. These authors reported that 100% (12/12) of the isolates from Cali carried the *bla*_KPC-2_ gene [7]. In another report by Rada et al. (2020), in which the dynamics of *bla*_KPC-2_ gene transmission through plasmids was studied in strains of *Enterobacteriaceae* in the city of Medellín, four isolates of the *E. cloacae* complex with ST456 were found to be circulating in three hospitals between 2013 and 2015 [18]. Additionally, Vanegas et al. (2016) studied the molecular epidemiology of Gram-negative bacilli resistant to carbapenems, isolated from a pediatric population in five hospitals in Medellín between 2012 and 2014. Out of the 24 *E. cloacae* complex isolates evaluated, one was found to carry the *bla*_KPC-3_ gene, although its ST was not reported [19]. Specifically, Colombia has been described as an endemic country for the gene that codes for KPC, with the KPC-2 and KPC-3 variants—those that circulate in the country—being more frequently detected in *K. pneumoniae* [11,20,21].

Regarding the STs detected in this study, 50% of the KPC-producing *E. cloacae* complex isolates belonged to ST510 and circulated in Cali from 2013 to 2018. These results agree with those of De la Cadena et al. (2017), who also reported ST510 as the most frequent ST found in the cities of Cali, Pereira and Medellín between 2009 and 2013. In particular, 80% of the *E. cloacae* complex isolates found in Cali carried the *bla*_KPC-2_ gene [7]. According to the MLST database of *E. cloacae*, apart from Colombia, ST510 has only been reported in Japan in 2016.

ST456 was found in 16.7% of the *E. cloacae* complex isolates carrying the *bla*_KPC-2_ gene, which circulated in Cali in 2017 and 2018. These results coincide with those reported by Rada et al. (2020), who found four *E. cloacae* complex isolates with ST456 carrying the *bla*_KPC-2_ gene in three hospitals in Medellín from 2013 to 2015 [18]. ST456 has been previously reported in a *E. cloacae* complex isolate carrying the *bla*_KPC-2_ gene in Norway in 2017 [22] and, according to the MLST database of *E. cloacae*, in other countries such as India (2013), Ghana (2015), Togo (2016) and the United States (2016 and 2018).

This is the first study reporting the presence of ST45, ST513 and ST1483 in Colombia. ST45 was found in 16.7% of the isolates circulating in Cali in 2017. This ST has been previously reported in Spain, in an *E. cloacae* isolate carrying the *bla*_OXA-48-like_ gene [23], and in China, in three CTXM-9- and SHV-12-producing isolates [24]. According to the MLST database of *E. cloacae*, ST45 has also been reported in Japan in 2013.

ST513 was found in 8.3% of the *E. cloacae* complex isolates carrying the *bla*_KPC-2_ gene and circulated in Cali in 2017. ST513 has been previously reported in Vietnam, in a colistin-resistant *E. cloacae* isolate that carried the *bla*_NDM-1_ gene and was isolated from a male patient in 2010 [25]. According to the MLST database of *E. cloacae*, ST513 has also been reported in Japan in 2016. Finally, ST1483 was found in 8.3% of the *E. cloacae* complex isolates carrying the *bla*_KPC-2_ gene, which circulated in Cali in 2014 and, according to the MLST database of *E. cloacae*, has also been reported by the Pasteur Institute of Guadeloupe in 2020.

The analysis of demographic data indicated that the KPC-producing *E. cloacae* complex mostly affected males (83%) and the elderly (32%), causing bloodstream infections (BSIs) (42%). In particular, BSIs are frequently acquired in hospital facilities and are considered a serious clinical condition that can worsen the prognosis of sepsis, leading to extended hospital stay, higher care costs, as well as increased morbidity and mortality [26]. According to the World Health Organization, 8.7% of nosocomial infections correspond to bacteremia and have gained importance in Europe, North America and Latin America, because they are usually caused by Gram-negative bacteria resistant to the available antibiotics [27]. For this reason, different antibiotics have been used to treat these microorganisms, including ceftazidime/avibactam which has shown activity against *Enterobacteriaceae* isolates producing carbapenemases from classes A and D, turning into a highly useful tool for the management of infections by multidrug-resistant *Enterobacteriaceae* [28]. However, resistance to this antibiotic has already been reported in class C β-lactamases [29] and KPC-producing *Enterobacteriaceae* [30]. Due to this, the therapeutic option will depend on the sensitivity profile of the isolate. This was the case of a 52-year-old female with infected arthritis of the right shoulder, whose joint aspirate culture showed a cefazolin-resistant *E. cloacae*. She was treated with levofloxacin, and she stopped experiencing shoulder swelling and severe pain [31]. Precisely, because sometimes there are few therapeutic options available to treat infections, in vitro studies using essential oils from plants have shown to have activity against multidrug-resistant *Enterobacteriaceae* isolates [32,33], which would be a basis for the development of new and effective antibacterial treatments.

In Colombia, bacterial isolates carrying the genes coding for metallo-beta-lactamases *bla*_VIM_ [34,35,36,37] and *bla*_NDM_ [38,39,40,41] have been reported. However, none of these genes have been previously found in *E. cloacae* isolates, which is in line with the results obtained in this study. This is also true for the *bla*_OXA-48-like_ gene, which was found for the first and only time in 2016 in Medellín, in an elderly patient infected with OXA-48-producing *K. oxytoca* [42], but not in the *E. cloacae* complex.

It is worth noting that none of the evaluated genes was detected in 57% of the isolates included in this study. Nonetheless, they were resistant to cephalosporins and at least to one carbapenem, which indicates that there is some other mechanism causing the resistance phenotype. Other carbapenemase not tested in this study, such as IMP or alteration or loss of nonspecific porins and hyper production of intrinsic, chromosomally encoded AmpC-type beta-lactamases, could cause such resistance [20]. Additional studies are required to elucidate these mechanisms.

The results show that, in addition to *K. pneumoniae* and *E. coli*, there are other *Enterobacteriaceae* such as KPC-2-producing *E. cloacae* complex, which are emerging as opportunistic pathogens resistant to carbapenems in Cali, Colombia. Therefore, it provides valuable information to further reinforce the epidemiological surveillance in the municipality of Santiago de Cali. Additionally, it was observed that some lineages are maintained over time not only in the region, but also in the country. This may be due to the referral of patients, which in turn makes the spread of these resistance mechanisms possible. It is crucial to pursue the molecular characterization of this bacterial species in different hospitals in the Department of Valle del Cauca and the rest of Colombia to design effective prevention and epidemiological surveillance strategies at the local, regional, and national levels. This will allow the establishment and implementation of institutional policies for the rational use of antibiotics in the hospitals of Colombia to prevent the spread of KPC-producing bacteria to all health institutions in the country.

## 4. Materials and Methods

### 4.1. Selection Criteria for E. cloacae Complex Isolates and Antibiotic Sensitivity Tests

In this retrospective and cross-sectional study, twenty-eight *E. cloacae* complex clinical isolates were sampled from 2011 to 2018. All isolates showing a phenotypic profile of total or intermediate resistance to third and fourth generation cephalosporins and to at least one carbapenem were selected according to the Clinical and Laboratory Standards Institute 2018 criteria [43]. Each of the clinical laboratories at the hospitals that participated in the study performed the identification and antibiotic sensitivity test using the automated system VITEK^®^ (BioMérieux, Marcy l’E’toile, France).

### 4.2. Detection of Genes Encoding Beta-Lactamase Enzymes

*E. cloacae* complex isolates were grown on MacConkey agar and incubated at 37 °C overnight. A single colony was resuspended in 100 µL of distilled water and bacteria were lysed in a water bath at 100 °C for 10 min. Cellular debris was removed by centrifugation at 13,000 rpm for 10 min and the supernatant was used as DNA template to perform polymerase chain reaction (PCR) [44]. To determine the quality of the cell lysate, we amplified a partial sequence of the 16S rRNA genes using universal primers U1 (5′-CCAGCAGCCGCGGTAATACG-3′) and U2 (5′-ATCGG(C/T)TACCTTGTTACGACTTC-3′), according with Lu et al., 2000 [44]. Then, the genes *bla*_KPC_, *bla*_NDM_, *bla*_VIM_ and *bla*_OXA-48-like_ were amplified by PCR using the primers shown in Table 4.

PCR was performed in an Eppendorf 950,000,040 Mastercycler thermal cycler using specific programs based on the hybridization temperature of the primers, as well as the size of the expected amplicons (Table 4). For this, 2X PCR 100 mixture (OPTBM-00006, CorpoGen, Bogotá, Colombia) was used following the manufacturer’s instructions. The reaction mix was prepared as follows: 1X master mix, 0.2 µM each of forward and reverse primers, <250 ng DNA and sterile water to make up the final volume to 25 µL. The following strains were used as positive controls for PCR: *K. pneumoniae* carrying the *bla*_KPC_ gene, *K. pneumoniae* carrying the *bla*_OXA-48-like_ gene, *P. aeruginosa* carrying the *bla*_VIM_ gene and *P. aeruginosa* carrying the *bla*_NDM_ gene. Sterile water was used to replace the volume corresponding to the DNA in negative controls.

Following PCR, the reaction products were purified using the commercial Qiaquick PCR Spin columns kit (Qiagen, Hilden, Germany) and were sequenced with the Sanger method in Macrogen, Korea. The sequences obtained were compared with the data from the National Center for Biotechnology Information database (https://blast.ncbi.nlm.nih.gov/Blast.cgi, accessed on 10 January 2021) and Beta-Lactamase Data Resources (https://www.ncbi.nlm.nih.gov/pathogens/beta-lactamase-data-resources/ accessed on 11 January 2021).

### 4.3. Molecular Genotyping of the KPC-Producing E. cloacae Complex Isolates

The genetic relationships between the KPC-producing *E. cloacae* complex isolates were determined using the PCR amplification technique multilocus sequence typing (MLST), according to Miyoshi-Akiyama et al. [49]. Following PCR, the amplified fragments were sequenced by Macrogen, Korea. Alleles and sequence types (STs) were assigned using the MLST website of *E. cloacae* (https://pubmlst.org/organisms/enterobacter-cloacae, accessed on 15 January 2021).

To establish the relationships between STs, the goeBURST algorithm [50] of the PHYLOViZ software (http://www.phyloviz.net/, accessed on 25 April 2021) was used.

## 5. Conclusions

This study identified the resistance mechanism and circulating genotypes of *E. cloacae* complex strains resistant to carbapenems isolated from five tertiary care hospitals in the municipality of Santiago de Cali. In 43% of the cases, the resistance to these antibiotics was due to the production of KPCs. Nonetheless, there are other mechanisms not detected in this study that confer resistance to beta-lactams. With regard to the circulating genotypes in the city, it was found that they are diverse and that ST510 and ST456 showed the highest proportion between 2011 and 2018. Moreover, these STs have been reported in other cities of the country with the greatest degree of spread. The other STs (ST45, ST513 and ST1483) are reported in this study for the first time in Colombia. The results obtained in this study demonstrate that, in addition to *K. pneumoniae* and *E. coli*, there are other *Enterobacteriaceae*, such as the *E. cloacae* complex carrying the variant of the *bla*_KPC-2_ gene, which could be emerging as carbapenem-resistant opportunistic pathogens in Cali, Colombia.

## Figures and Tables

**Table 1:** Antibiotic susceptibility profile and demographic data of the *E. cloacae* complex isolates included in this study.

Key	Year Isolate	Ceftazidime	Cefepime	Ceftriaxone	Ertapenem	Meropenem	Origin	Infection	Age (Years)	Gender
1416	2011	>64	4	>64	4	<1	Clinic 1	Urine	81	F
1666	2012	16	8	>64	>8	1	Clinic 1	Bloodstream	9	M
2072	2012	16	2	>64	4	>16	Clinic 1	Urine	41	M
1364	2013	>64	>64	>64	>8	>16	Clinic 1	Bloodstream	49	M
2205	2013	>64	2	>64	4	2	Clinic 1	Urine	6	M
2249	2013	16	2	4	2	1	Clinic 1	Urine	84	F
1359	2014	>64	>64	>64	>8	>16	Clinic 1	Skin	93	F
4290	2014	32	32	>64	>8	>16	Clinic 1	Bloodstream	23	M
4347	2014	>64	>64	>64	>8	>16	Clinic 1	Urine	67	M
4730	2015	>64	>64	>64	>8	>16	Clinic 1	Ulcer	20	F
5047	2016	16	>64	>64	>8	>16	Clinic 1	Urine	17	M
5227	2017	>64	>64	>64	>8	>16	Clinic 1	Wound	58	M
382	2017	>64	2	>64	4	<1	Public	Bloodstream	76	F
474	2017	32	16	32	4	2	Public	Peritoneal fluid	22	M
381	2017	16	16	>32	4	1	Public	Bloodstream	74	M
713	2017	16	4	>4	>1	8	Public	Peritoneal fluid	54	M
332	2017	16	2	>64	2	2	Clinic 2	Bloodstream	21 days	M
331	2017	>64	>64	>64	2	<1	Clinic 2	Bloodstream	30	M
333	2017	>64	>64	>64	>8	>16	Clinic 2	Bloodstream	47 days	M
330	2017	>64	>64	>64	>8	>16	Clinic 2	Bloodstream	12 days	M
305	2017	>64	>64	>64	>8	>16	Clinic 3	Sore	30	M
31	2017	>64	2	>64	4	2	Clinic 3	Wound	20	F
2	2018	>64	>64	>64	>8	>16	Clinic 4	Urine culture	70	M
1,510,006	2018	>64	>64	>64	>8	>16	Public	Catheter	62	M
6	2018	>64	>64	>64	>8	>16	Public	Bloodstream	62	M
5385	2018	>64	>64	>64	>8	>16	Clinic 1	Sore	55	M
5438	2018	16	2	≥64	4	1	Clinic 1	Urine	18	M
5521-12	2018	16	2	≥64	4	1	Clinic 1	Urine	1	F

**Table 2:** Genetic relationship of *E. cloacae* complex isolates carrying the *bla*_KPC_ variant.

Key	Allele	ST	*dna*A	*fus*A	*gyr*B	*leu*S	*pyr*G	*rpl*B	*rpo*B
1364	*bla*_KPC-2_	510	4	4	4	209	171	4	115
1359	*bla*_KPC-2_	1483	376	21	9	44	45	4	33
4290	*bla*_KPC-2_	510	4	4	4	209	171	4	115
4730	*bla*_KPC-2_	510	4	4	4	209	171	4	115
5227	*bla*_KPC-2_	510	4	4	4	209	171	4	115
474	*bla*_KPC-2_	513	171	1	190	168	1	22	113
333	*bla*_KPC-2_	45	4	4	14	6	39	4	6
330	*bla*_KPC-2_	45	4	4	14	6	39	4	6
305	*bla*_KPC-2_	456	149	44	61	180	152	1	1
2	*bla*_KPC-2_	456	149	44	61	180	152	1	1
1,510,006	*bla*_KPC-2_	510	4	4	4	209	171	4	115
6	*bla*_KPC-2_	510	4	4	4	209	171	4	115

Abbreviations: Sequence type (ST).

**Table 3:** Demographic characteristics corresponding to the *E. cloacae* complex isolates carrying the *bla*_KPC_ gene.

Characteristic	% of Isolates
**Gender**	
Female	17% (2/12)
Male	83% (10/12)
**Age**	
Newborn (0–30 days)	8% (1/12)
Infant (1–12 months)	8% (1/12)
Teenagers (12–20 years)	8% (1/12)
Young adult (21–40 years)	16% (2/12)
Middle adult (41–60 years)	24% (3/12)
Elderly (>60 years)	32% (4/12)
**Infection site**	
Bloodstream	42% (5/12)
Skin	8.3% (1/12)
Ulcer	8.3% (1/12)
Wound	8.3% (1/12)
Peritoneal fluid	8.3% (1/12)
Sore	8.3% (1/12)
Urine culture	8.3% (1/12)
Catheter	8.3% (1/12)

**Table 4:** Primers used for polymerase chain reaction of the carbapenem-resistant *E. cloacae* complex isolates.

Primer Name	Primer Sequence	Product Size (bp)	Annealing Temperature (°C)	References
KPC F	5-TGTCACTGTATCGCCGTC-3	894	53	Yigit y col., 2001 [45]
KPC R	5-CTCAGTGCTCTACAGAAAACC-3			
NDM F	5-ATGGAATTGCCCAATATTATGC-3	813	52	Liu y col., 2013 [46]
NDM R	5-TCAGCGCAGCTTGTCGGCCAT-3			
VIM F	5-GTCTATTTGACCGCGTC-3	775	52	Toleman y col., 2002 [47]
VIM R	5-CTACTCAACGACTGAGCG-3			
OXA-48 F	5-TATATTGCATTAAGCAAGGG-3	848	56	Poirel y col., 2011 [48]
OXA-48 R	5-CACACAAATACGCGCTAACC-3			

## Data Availability

The data used to support the findings of this study are included within the article and are available from the corresponding author upon request.
